# Supplementary material for: The Association of R-Loop Binding Proteins Subtypes with CIN Implicates Therapeutic Strategies in Colorectal Cancer
Source: Cancers (Basel). 2022 Nov 15;14(22):5607. doi: 10.3390/cancers14225607 (PMC9688457; doi:10.3390/cancers14225607)
Supplement: Supplementary file 1 [file cancers-14-05607-s001.zip › cancers-1997776-supplementary.pdf]

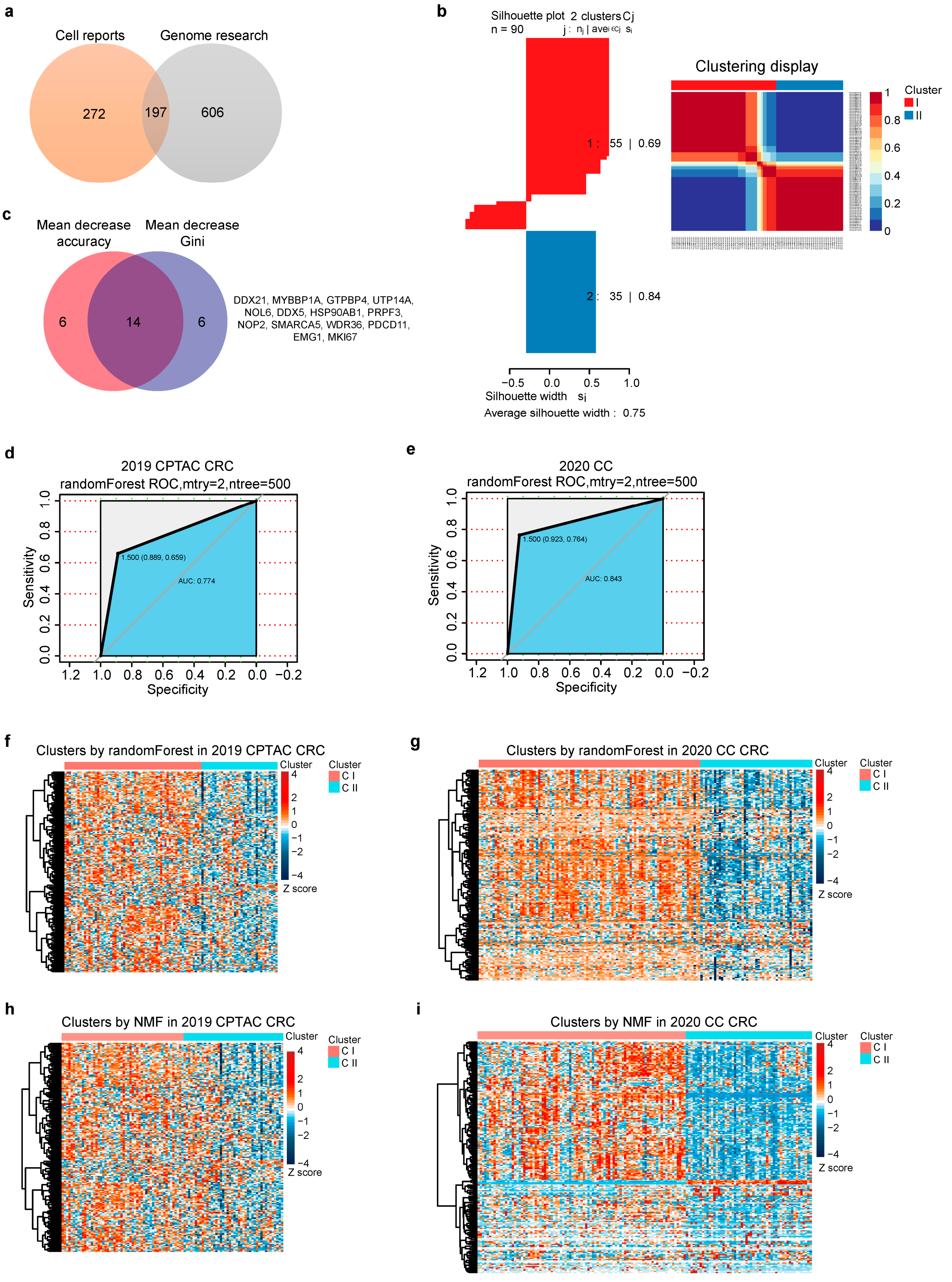

Figure S1. Proteomics-based molecular subtypes by the random forest model in 2019 and 2020 CRC data-bases.

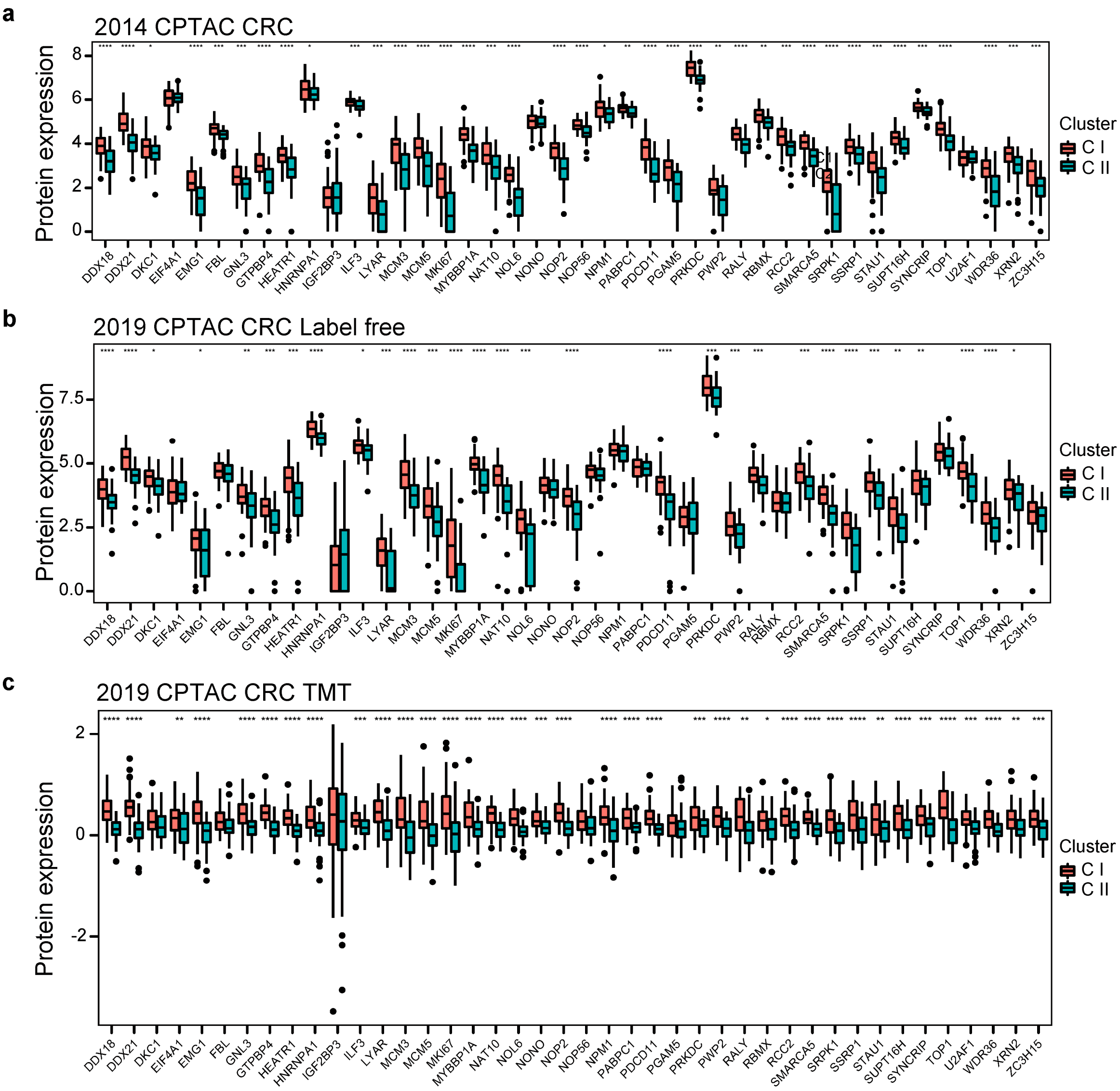

Figure S2. Proteomics-based clustering of RLBPs expression in CRC patients.

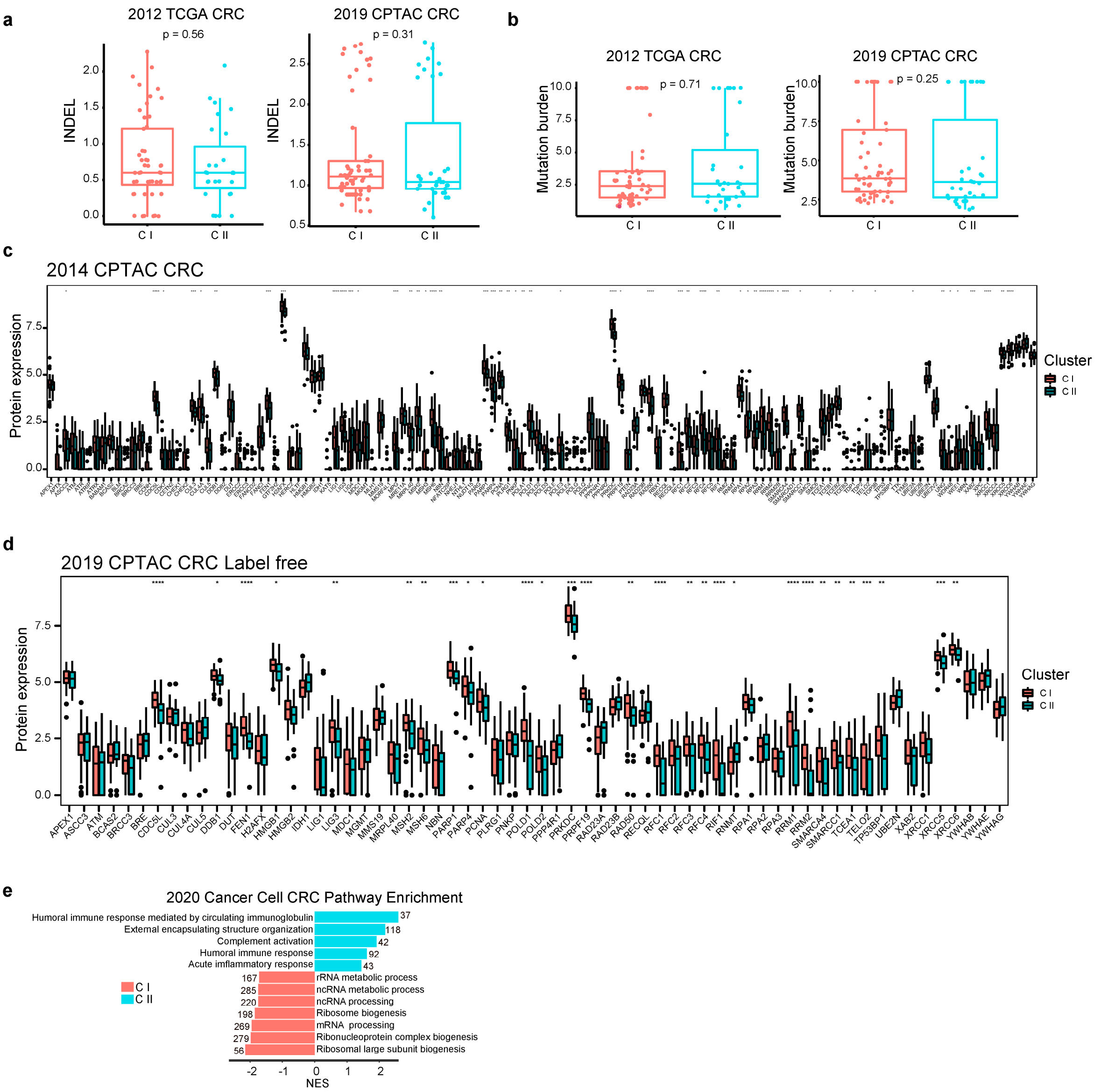

Figure S3. Landscape of pathway enrichment and DDR genes expression in two clusters.

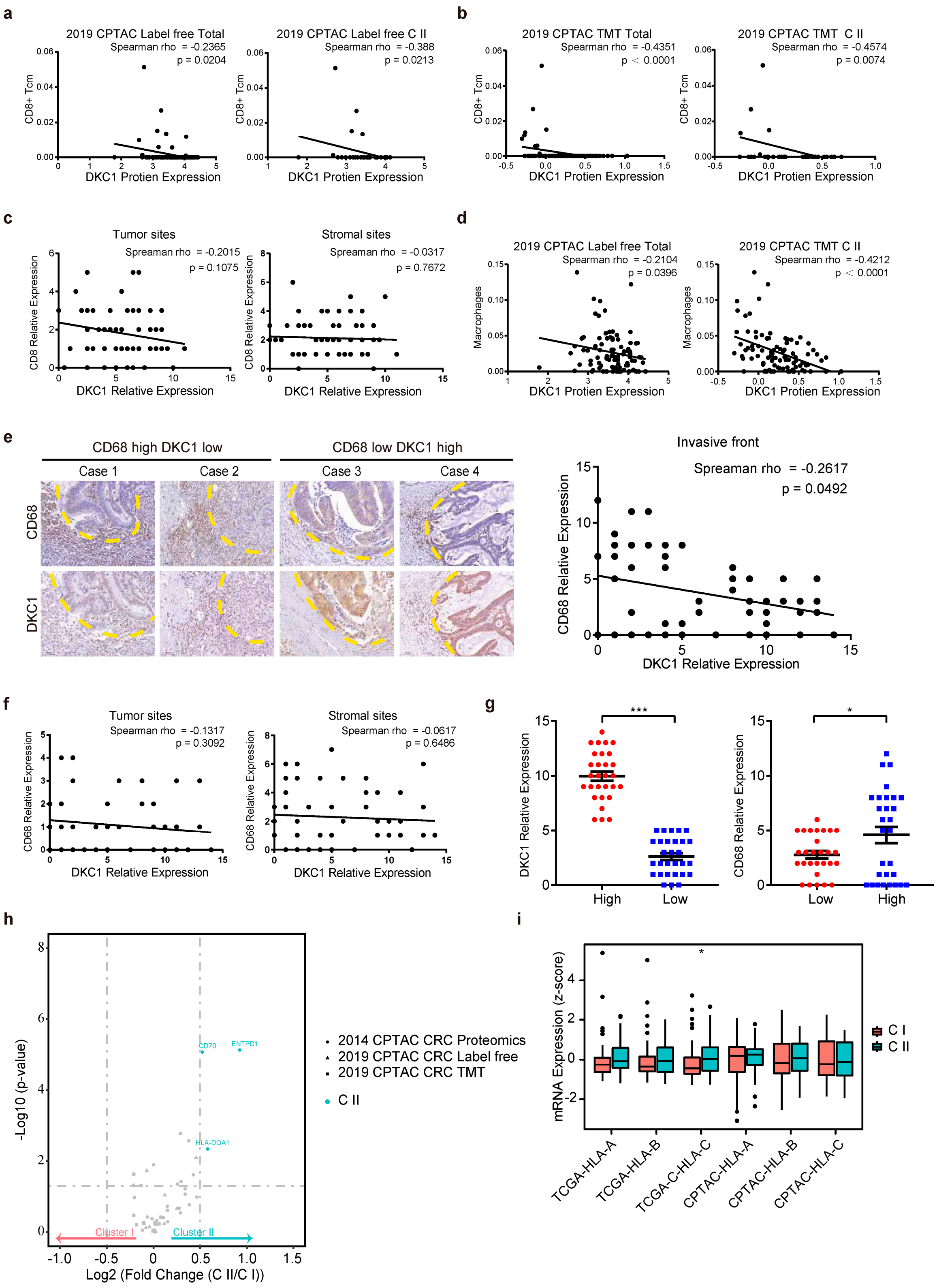

Figure S4. The correlation of RLBPs with immunomodulatory factors.

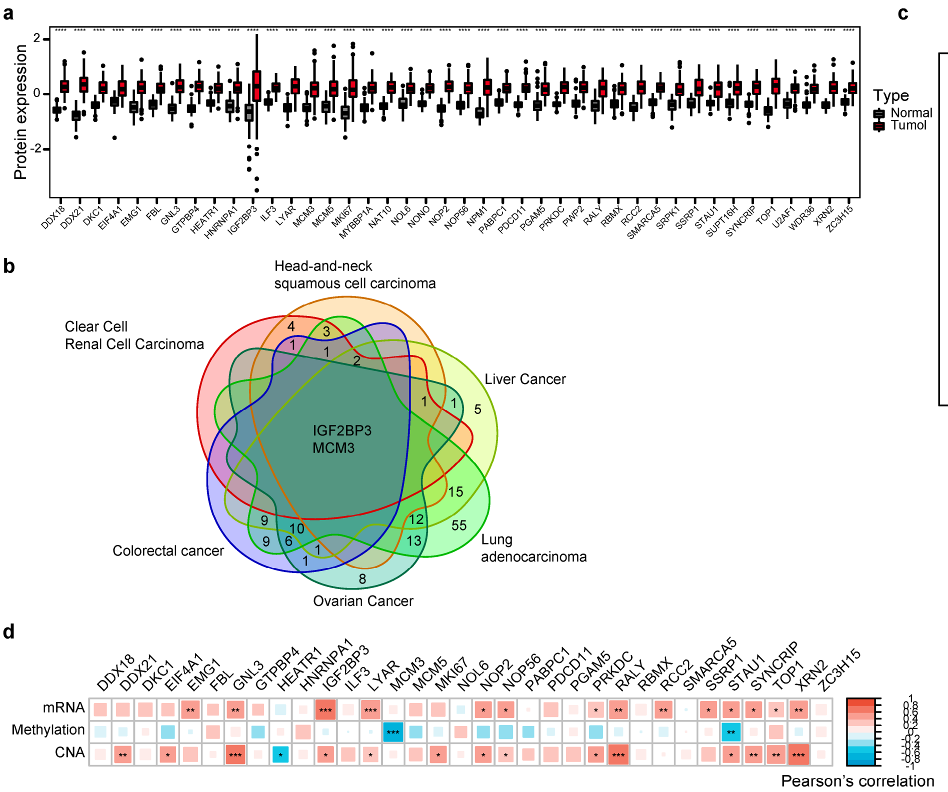

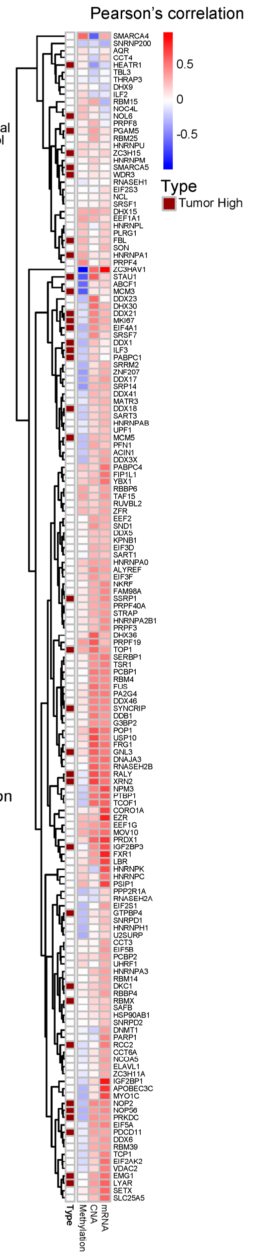

e

Cancer databases (TCGA/CPATC)

	Pearson r	p value	Numbers	Percentage
CNA	r > 0.3	p < 0.05	8/42	19.0%
DNA Methylation	r < -0.3	p < 0.05	3/42	7.1%
mRNA	r > 0.3	p < 0.05	14/42	33.3%

Cell databases (CCLE)

	Pearson r	p value	Numbers	Percentage
CNA	r > 0.3	p < 0.05	14/33	42.4%
DNA Methylation	r < -0.3	p < 0.05	2/33	6.0%
mRNA	r > 0.3	p < 0.05	14/33	42.4%

Figure S5. The RLBPs are affected by CNA, DNA methylation (promoter CpG clusters) or transcriptional regulations.

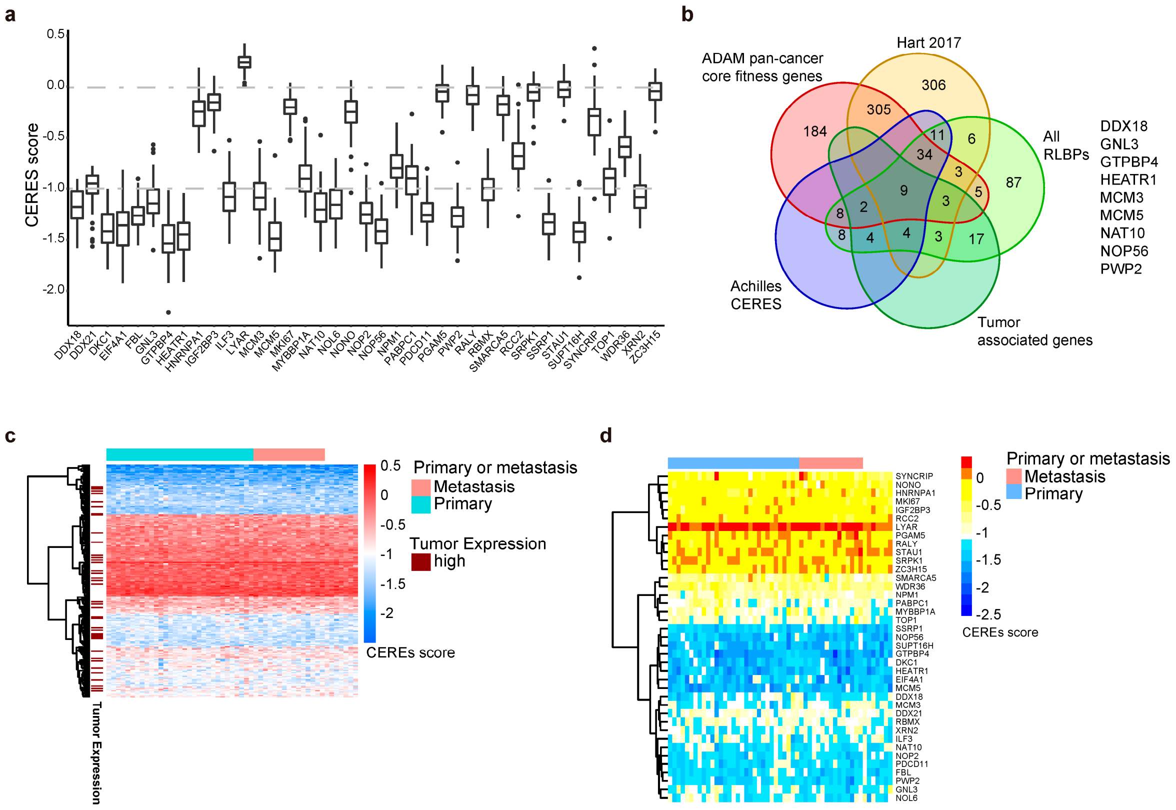

Figure S6. RLBPs serve as potential therapeutic targets.

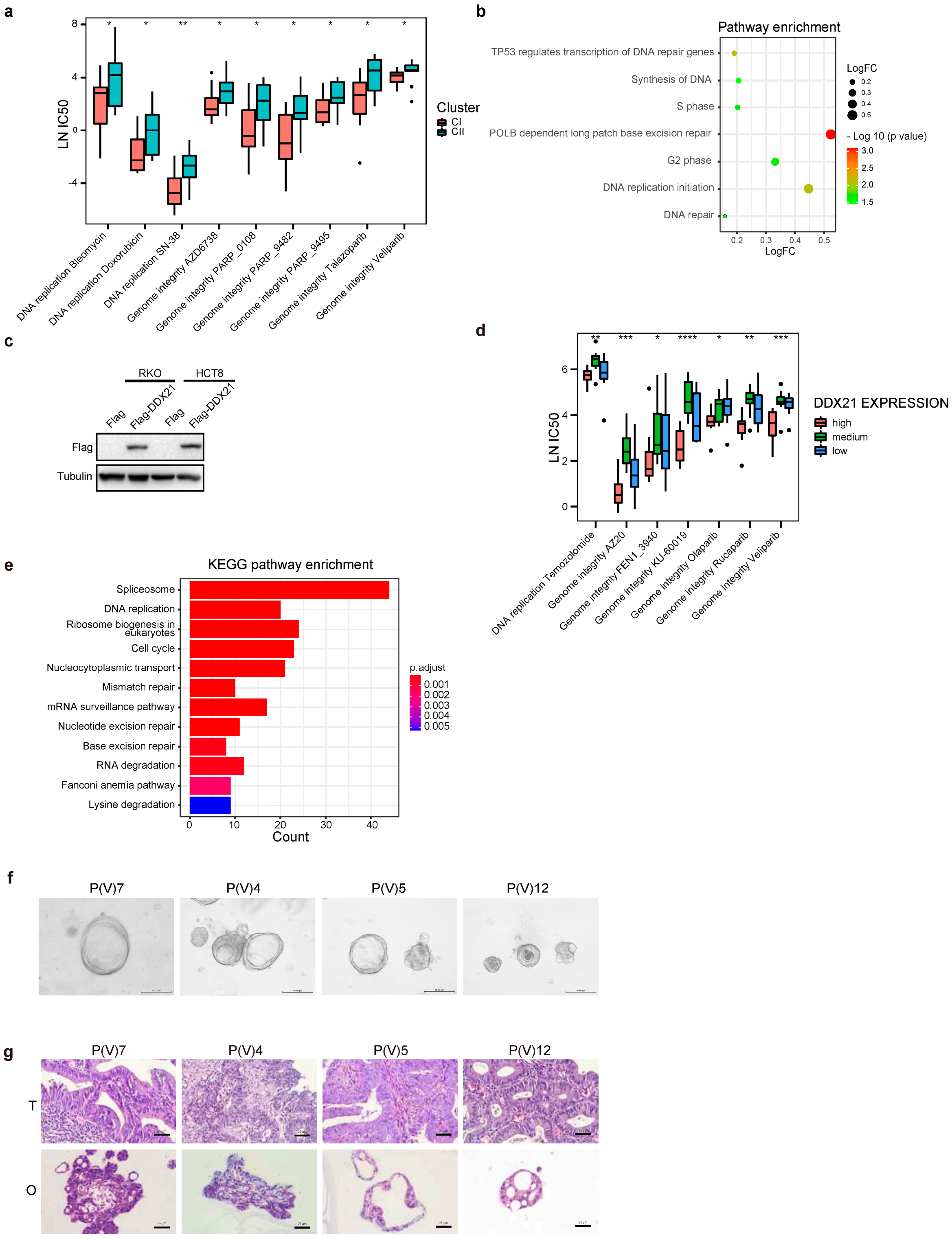

Figure S7. The Drug sensitivity analysis in cells with differential RLBP expression.
